# Prevalence and Prognostic Impact of Hyponatremia in Guillain-Barré Syndrome: A Systematic Review and Meta-Analysis

**DOI:** 10.7759/cureus.67215

**Published:** 2024-08-19

**Authors:** Yasemin Ekmekyapar Fırat, Zeynep Karaoglu Akıncı, Buse Gül Belen, Cansu Gülcihan Türkok, Sevki Sahin, Sibel Karsidag

**Affiliations:** 1 Neurology, Sanko University School of Medicine, Gaziantep, TUR; 2 Neurology, Sultan 2. Abdulhamid Han Training and Research Hospital, University of Health Sciences, Istanbul, TUR; 3 Neurology, University of Health Sciences, Sancaktepe Research and Training Hospital, Istanbul, TUR

**Keywords:** acute inflammatory demyelinating polyneuropathy, prognosis, meta-analyses, hyponatremia, guillain barre sydrome

## Abstract

This study aims to systematically review the existing literature and perform a meta-analysis to evaluate the prevalence of hyponatremia among Guillain Barre Syndrome (GBS) patients and its relationship with disease prognosis. We comprehensively searched PubMed, Embase, Medline, Web of Science, Science Direct, and the Cochrane Library databases from 1995 to 2024 for observational studies on the prevalence of hyponatremia in GBS. The meta-analysis followed the Preferred Reporting Items for Systematic Reviews and Meta-Analyses (PRISMA 2020) guidelines. Heterogeneity among the included studies was calculated with the I^2^ for each analysis. We used the Comprehensive Meta-Analysis software (Version 3.3.070; Biostat, Englewood, USA). Eight observational studies met our inclusion criteria. The meta-analysis showed that the pooled prevalence of hyponatremia among GBS patients was 12% (95% CI: 0.107-0.149). The results exhibited high heterogeneity (I² = 99%), indicating significant variability among the studies. Hyponatremia rates reported in these eight studies ranged from 11.5% to 48% in GBS patients. The prevalence of hyponatremia was found to be 12% in GBS patients, which is relatively lower compared to some reports. Hyponatremia was found to be associated with prolonged hospital stay, mortality, and mechanical ventilation as poor prognostic factors. Further prospective studies are needed to elucidate the mechanisms underlying hyponatremia in GBS and to develop targeted interventions to address this issue.

## Introduction and background

Guillain-Barre Syndrome (GBS) is the most common cause of acute flaccid paralysis worldwide, with an incidence rate ranging from 0.6 to 4 per 100,000 individuals annually [1]. Characterized by the rapid onset of muscle weakness and areflexia, GBS can lead to significant morbidity and mortality. Clinical features of GBS include symmetrical limb weakness, decreased or absent deep tendon reflexes, albuminocytologic dissociation in cerebrospinal fluid, and distinct electrodiagnostic findings [1]. GBS is believed to have an immune-mediated etiology, often triggered by infections or vaccinations [2, 3].

Despite advancements in understanding the pathogenesis and treatment of GBS, certain complications, such as hyponatremia, can impact the prognosis of affected individuals. Hyponatremia, defined as a serum sodium level below 135 mmol/L, is a common electrolyte disturbance in hospitalized patients. In the context of GBS, hyponatremia can arise due to various mechanisms, including the syndrome of inappropriate secretion of antidiuretic hormone (SIADH), the effects of intravenous immunoglobulin (IVIg) therapy, and renal salt wasting associated with autonomic dysfunction [4-6]. It is appropriate to include electrolytes among the tests in patients with GBS. The association between hyponatremia and GBS has been reported previously in small-size studies. Previous studies have suggested that hyponatremia in GBS patients is associated with more severe disease manifestations and poorer outcomes [4-6].

Given the potential impact of hyponatremia on the clinical course of GBS, it is crucial to understand its prevalence and prognostic significance. This study aims to systematically review the existing literature and perform a meta-analysis to evaluate the prevalence of hyponatremia in GBS patients and its relationship with disease prognosis. By consolidating data from multiple studies, we hope to provide a comprehensive assessment of the implications of hyponatremia in GBS and inform clinical practice.

## Review

Materials and methods

​​The meta-analysis was carried out in accordance with the Preferred Reporting Items for Systematic Reviews and Meta-Analyses (PRISMA 2020) [7]. Two authors (YEF and ZKA) independently conducted a comprehensive literature search in PubMed, Embase, Medline, Web of Science, Science Direct, and the Cochrane Library databases from 1995 to 2024. The search terms used were “Guillain-Barré syndrome,” “hyponatremia,” “inappropriate secretion of antidiuretic hormone,” and “SIADH.” Initially, keywords were given and the studies were selected with their titles and abstracts by two researchers (CGT and BGB). The other two authors (YEF and ZKA) independently evaluated the articles for suitability, and duplicated articles were removed.

English-language cohort and case-control studies evaluating hyponatremia in human patients diagnosed with GBS, including those comparing outcomes such as mortality, length of hospital stay, mechanical ventilation requirement, and severity scores between hyponatremic and normonatremic patients, were included.

The initial database search yielded 1219 articles. After removing duplicates and non-research articles, titles and abstracts were screened for relevance. Full texts of potentially eligible studies were then retrieved and assessed for inclusion based on the predefined criteria. Discrepancies were resolved by discussion and, if necessary, by consulting a third author (SK).

Data from the included studies were extracted independently by two authors (YEF and ZKA) using a standardized data extraction form. The following information was recorded: primary author, publication year, study location, study period, study design, number of participants, patient demographics (age and gender), criteria used for diagnosing hyponatremia, criteria for diagnosing GBS, and key outcomes (mortality, length of hospital stay, mechanical ventilation, and plasmapheresis status).

The quality of the included studies was evaluated using the Newcastle-Ottawa Scale (NOS) for observational studies. This scale assesses the quality of studies based on three domains: selection of study groups, comparability of groups, and ascertainment of outcomes. Each study was independently assessed by two reviewers (YEF and ZKA). In cases of disagreement, a third reviewer (SK) adjudicated.

We used the Comprehensive Meta-Analysis software (Version 3.3.070; Biostat, Englewood, USA) to assess the meta-analysis. Heterogeneity among the included studies was assessed using the I² statistic. The heterogeneity was larger than 50% between the studies, fixed effects model was used in order to calculate the pooled effect and its 95% confidence interval (CI). The odds ratios (OR) were determined using the Z test. Publication bias was evaluated using the Funnel graph, Egger's regression test, and Begg's rank correlation test.

Results

**Figure 1:**
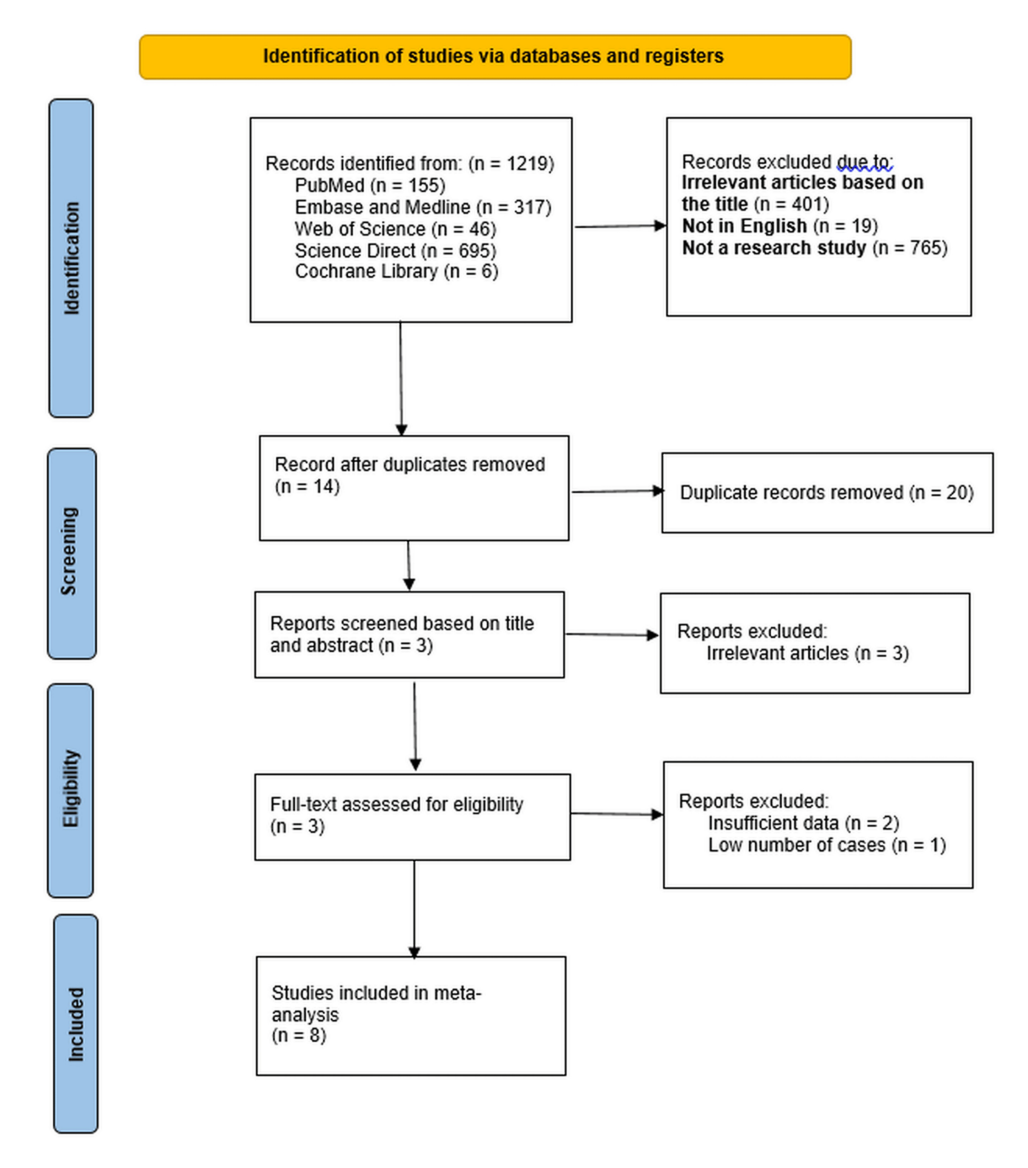
PRISMA flow diagram PRISMA: Preferred Reporting Items for Systematic Reviews and Meta-Analyses

An initial extensive database search yielded 1219 articles related to the topic. After removing irrelevant, duplicate, non-English, non-research articles, eight studies were found suitable for meta-analysis (Figure 1) [7]. A total of 55757 patients were evaluated for our quantitative analysis.

The baseline characteristics of the included studies are presented in Table 1.

**Table 1:** General characteristics of the studies included GBS: Guillain-Barré Syndrome

No	Authors	Location	Study period	Case ascertainment	Case definition	Hyponatremia criteria
1	Martinez-Pina et al [8]	Mexico City, Mexico	January 1, 2018, to December 2021	Observational, cross-sectional, comparative, and analytical study,	Ausbury criteria	Patients with serum so­dium levels < 135 meq/L on admission and, before ini­tiation of treatment (plasma exchanges or human im­munoglobulin) were classified as hyponatremia.
2	Colls [9]	New Zealand	1 July 1990 and 30 June 2000.	Retrospective review of medical discharge records	Asbury and Cornblath criteria	The serum sodium was documented in all patients on admission to hospital, before the initiation of treatment.
3	Gagliardi et al [10]	Milan, Italy	January 2010 to July 2020	Retrospective cohort study	Brighton criteria	Hyponatremia was defined as serum sodium levels < 135 mEq/L during hospitalization
4	Hiew et al [11]	Birmingham, UK	2007 and 2012	Retrospective review of medical discharge records		Hyponatraemia was defined as serum sodium levels ≤133 mmol/L occurring at any time during the first 3 weeks of admission.
5	Wang & Liu [12]	Shijiazhuang, China	2003 to 2012	Retrospective review of medical discharge records	GBS-consensus group of the Dutch Neuromuscular Research Support Centre	Hyponatremia is defined as serum sodium concentration below 135 mmol/L at nadir, including mild hyponatremia (130–135 mmol/L), moderate hyponatremia (125–130 mmol/L), and severe hyponatremia (<125 mmol/L).
6	Ng et al [13]	London, UK	January 1985 and December 1992	Retrospective review of medical discharge records	Asbury and Cornblath criteria	Hyponatraemia was considered to be significant when serum sodium was <133mmol/L (range 122-132 mmol/L) on more than two serial results.
7	Rumalla et al [14]	Kansas City, USA	2002 to 2011	Retrospective review of The Nationwide Inpatient Sample (NIS)	International Classification of Diseases, 9th Revision, (ICD-9-CM) code for GBS (357.0)	The standard hyponatremia was defined as less than or equal to 135 mmol/L using ICD-9-CM codes
8	Saifudheen et al [4]	Kerala, India.	January 2009 to April 2010	Prospective observational study	Asbury criteria	Serum sodium levels were recorded daily for initial 3 weeks. Syndrome of inappropriate secretion of antidiuretic hormone (SIADH) was diagnosed based on modified Bartter and Schwartz criteria.

The first study included was by Ng et al. (1995) [13]. It was shown that the prevalence of hyponatremia was 25.3% in 79 GBS patients who were admitted to the neurology intensive care unit. The second study included was by Colls et al. (2003), who found the prevalence of hyponatremia to be 31% in 84 GBS patients [9]. However, in 12 of the patients, the hyponatremia was pseudohyponatremia secondary to IVIG use. The third study was by Wang et al. (2015), which they reported the prevalence of hyponatremia as 21.5% in 455 GBS patients [12].

In the GBS series of Rumalla et al. [14] in 2017, hyponatremia was recorded at a rate of 11.8% in 54778 patients. In their prospective study published in 2011, Saifudheen et al. [4] stated that the rate of hyponatremia due to inappropriate antidiuretic hormone release was 48% in 50 GBS patients. In the study of Hiew et al. [11] published in 2016, hyponatremia was detected in 37.5% of 48 patients, but it was stated that hyponatremia was due to immunoglobulin treatment in 10 of 18 patients. Gagliardi et al. [10] described hyponatremia in 25.5% of 51 GBS patients in their retrospective study. Martinez-Pina et al. [8] reported a 14.6% rate of hyponatremia in 212 GBS patients in their observational cross-sectional study published in 2023.

The Prevalence of Hyponatremia in GBS Patients

**Figure 2:**
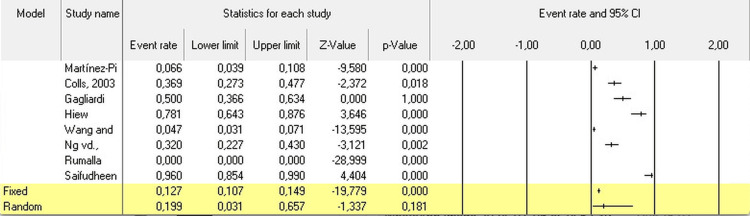
Meta-analysis of hyponatremia in GBS patients Martinez-Pi [8], Colls, 2003 [9], Gagliardi [10], Hiew [11], Wang [12], Ng [13], Rumalla [14], Saifudheen [4]

The meta-analysis revealed a pooled prevalence of 12% for hyponatremia among GBS patients (95% CI: 0.107-0.149). The results exhibited high heterogeneity (I² = 99%), indicating significant variability among the studies (Figure 2).

Risk of Bias Assessment

**Figure 3:**
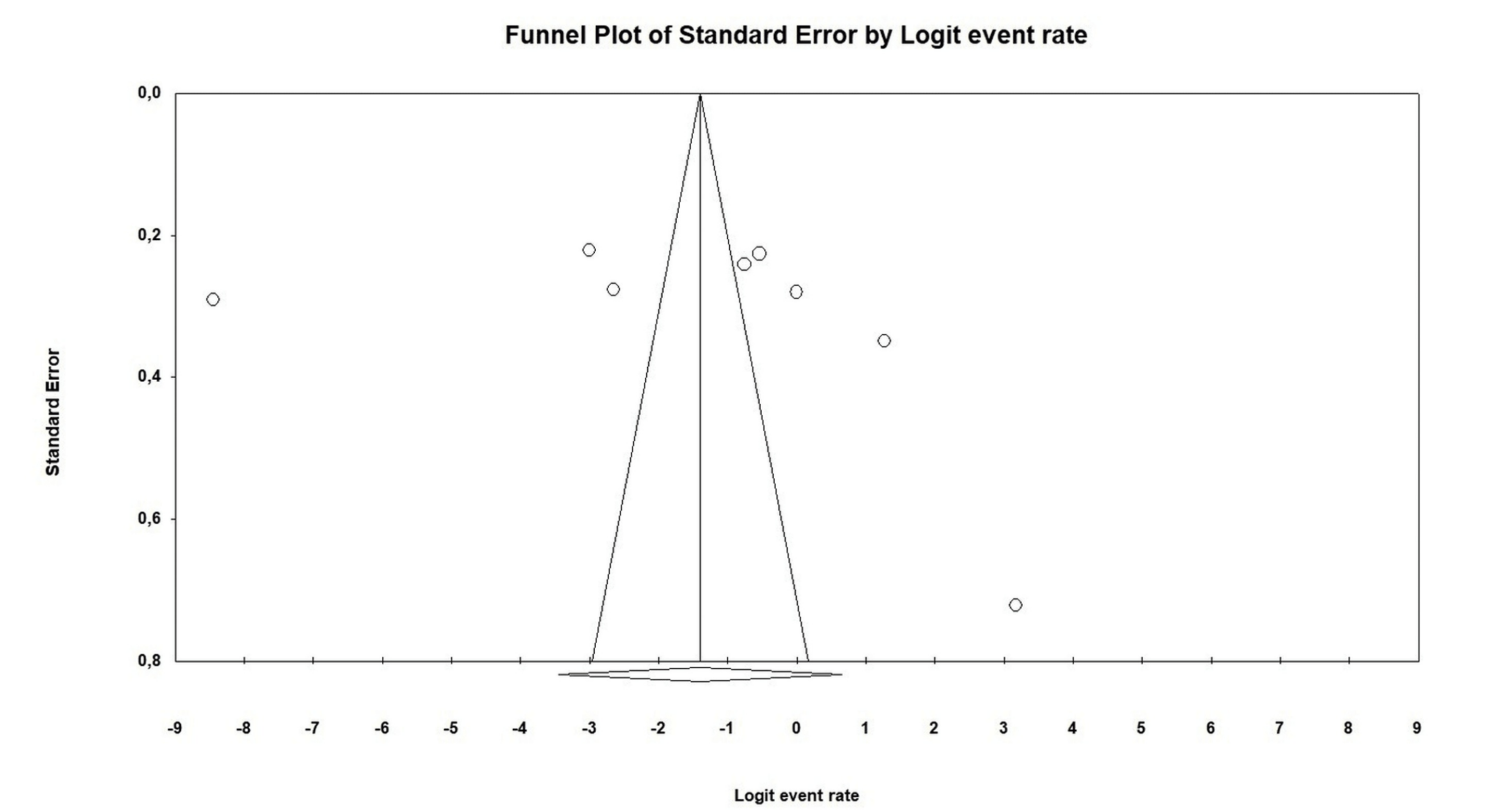
Funnel plot of studies Egger test (p=0.61) and Begg’s test (p=0.32) showed no publication bias.

Doi plot analysis showed that the results were symmetrical, indicating that they were not affected by publication bias (Figure 3).

Metaregression

Univariate metaregression analysis for mortality, hospital stay, and mechanical ventilation did show a significant association with the effect size of the study. Age, bulbar weakness, sex, and plasmapheresis did not show sufficient effect size (Table 2).

**Table 2:** Results of the univariate metaregression analysis

Covariate	Coefficient (95 %CI)	p
Mortality	-0.3469 (-0.6250 to 0.0688)	0.0145
Hospital stay	0.5388 (0.0713 to 1.0063)	0.0239
Mechanical ventilation	-0.0900 (-0.1642 to -0.0158)	0.0175

Discussion

Hyponatremia appears to be a poor prognostic indicator in GBS patients, as suggested by multiple retrospective cohort studies. Some studies have reported associations between hyponatremia and severe clinical features such as bulbar weakness, the requirement for mechanical ventilation, prolonged hospital stay, lower Medical Research Council (MRC) score, higher scores on the Hughes scale, and increased mortality rates [4, 9, 11, 12]​​.

Our systematic review included eight studies that addressed the incidence and correlates of hyponatremia in GBS. These studies varied in design, with seven being retrospective and one prospective, and reported hyponatremia rates ranging from 11.5% to 48% among GBS patients. The variance in hyponatremia prevalence could be attributed to several factors, including differences in diagnostic criteria, patient populations, and treatment modalities.

Our meta-analysis revealed that the prevalence of hyponatremia in GBS patients was 12 % (95% CI: 0.107-0.149). The results were markedly heterogeneous, as indicated by an I² of 99%. Despite this heterogeneity, the analysis demonstrated that GBS patients with hyponatremia tended to have longer hospital stays, higher mortality, and need for mechanical ventilation compared to those without hyponatremia. This finding underscores the need for vigilant monitoring and management of sodium levels in GBS patients to potentially mitigate the adverse impacts on hospital duration and overall prognosis​.

One significant factor contributing to hyponatremia in GBS patients is IVIg therapy, commonly used in the treatment of GBS [5]. IVIg can cause a notable decrease in serum sodium levels, a condition termed pseudohyponatremia [15]. This phenomenon is explained by the dilutional effect of the IVIg preparation, which contains high protein content. Additionally, plasmapheresis, another treatment modality for GBS, has been linked to elevated antidiuretic hormone (ADH) levels, contributing to the development of hyponatremia [16].

Another significant mechanism underlying hyponatremia in GBS is the syndrome of inappropriate antidiuretic hormone secretion (SIADH). SIADH is characterized by excessive release of ADH, leading to water retention and dilutional hyponatremia. The pathophysiology of SIADH in GBS may involve the dysregulation of osmoreceptors in the hypothalamus. Additionally, the release of vasopressin may be mediated by interleukin-6 (IL-6) in the terminal lamina [17]. Renal salt wasting syndrome, another potential cause, is thought to arise from inadequate secretion of brain natriuretic peptide, leading to dysautonomia in GBS [6]​.

Limitations

This meta-analysis has several limitations. The high heterogeneity among the included studies, with an I² of 99%, suggests significant variability in study designs, populations, and methodologies. Additionally, the majority of included studies were retrospective, which could introduce bias. The differences in criteria for diagnosing hyponatremia and the variable timing of serum sodium measurements further complicate the interpretation of results​.

However, the strengths of this analysis include a comprehensive search strategy and the inclusion of a large number of patients, which enhances the generalizability of the findings. Moreover, the use of robust statistical methods to assess heterogeneity and publication bias adds to the reliability of the conclusions drawn. Doi plot analysis showed that the results were symmetrical, indicating that they were not affected by publication bias​​.

## Conclusions

In conclusion, hyponatremia is a common and significant prognostic factor in GBS patients. Its presence is associated with more severe disease manifestations and longer hospital stays. Clinicians should be aware of the potential for hyponatremia in GBS patients and consider regular monitoring and appropriate management strategies to improve outcomes. Prospective studies are warranted to further elucidate the mechanisms underlying hyponatremia in GBS and to develop targeted interventions to address this issue.
